# PPARδ attenuates hepatic steatosis through autophagy-mediated fatty acid oxidation

**DOI:** 10.1038/s41419-019-1458-8

**Published:** 2019-02-27

**Authors:** Lei Tong, Long Wang, Shuangshuang Yao, Lina Jin, Jian Yang, Yifei Zhang, Guang Ning, Zhiguo Zhang

**Affiliations:** Department of Endocrinology and Metabolism, Shanghai Clinical Center for Endocrine and Metabolic Diseases, Shanghai Institute of Endocrine and Metabolic Diseases, Ruijin Hospital, China National Research Center for Metabolic Diseases, Shanghai Jiao Tong University School of Medicine, 200025 Shanghai, China

## Abstract

Peroxisome proliferator-activated receptor δ (PPARδ) belongs to the nuclear receptor family and is involved in metabolic diseases. Although PPARδ is known to attenuate hepatic lipid deposition, its mechanism remains unclear. Here, we show that PPARδ is a potent stimulator of hepatic autophagic flux. The expression levels of PPARδ and autophagy-related proteins were decreased in liver tissues from obese and ageing mice. Pharmacological and adenovirus-mediated increases in PPARδ expression and activity were achieved in obese transgenic db/db and high fat diet-fed mice. Using genetic, pharmacological and metabolic approaches, we demonstrate that PPARδ reduces intrahepatic lipid content and stimulates β-oxidation in liver and hepatic cells by an autophagy–lysosomal pathway involving AMPK/mTOR signalling. These results provide novel insight into the lipolytic actions of PPARδ through autophagy in the liver and highlight its potential beneficial effects in NAFLD.

## Introduction

Non-alcoholic fatty liver disease (NAFLD) is recognised as the leading cause of chronic liver disease in adults and children^1^, with histological characteristics ranging from simple fatty liver (steatosis) to non-alcoholic steatohepatitis (NASH) and cirrhosis; some cases even develop into end-stage liver disease and hepatocellular carcinoma^2,3^. NAFLD appears to be highly associated with obesity and diabetes. NAFLD is characterised by the progressive accumulation of triglycerides (TGs) in hepatocytes, which could result from increased free fatty acid (FFA) uptake into the liver, impaired lipid catabolism or enhanced de novo lipogenesis^4,5^.

In recent decades, there have been tremendous advances in understanding the regulatory effect of autophagy on hepatic lipid metabolism. Autophagy is an evolutionarily conserved physiological process that represents a system of bulk protein degradation aimed at the removal and breakdown of cellular components (organelles and proteins) during starvation, thereby redistributing nutrients to maintain cellular energetic balance^6^. It also plays a critical role in eliminating damaged proteins and organelles^7^. Deficiencies in autophagy flux are closely related to the development of hepatic steatosis. Autophagy is supposed to break down intracellular lipids in hepatocytes through a lysosomal degradation pathway and therefore may regulate the development of hepatic steatosis^5,8,9^.

Peroxisome proliferator-activated receptor (PPAR) agonists are well established in therapeutic areas related to lipid and glucose metabolism, such as T2DM, obesity and dyslipidaemia^10–12^. PPARα is one of the most abundantly expressed nuclear receptors in the liver^12,13^. PPARα and its agonists have hepatoprotective effects in rodent models of NAFLD/NASH. However, fibrates and other available PPARα agonists have shown no beneficial effects on steatosis in human studies^14^. PPARα expression is low in the human liver relative to the rodent liver, and this expression level progressively decreases as NASH progresses in humans, which may explain the contradictory results of early PPARα agonists in randomised clinical trials^13,15,16^. PPARδ is ubiquitously expressed and has been implicated in lipid metabolism and energy homoeostasis in various organs, including the liver^16^. Moreover, in recent clinical studies that included overweight patients with mixed dyslipidaemia, there was a reduction in hepatic fat content upon treatment with PPARδ agonists^17,18^. However, the exact mechanism by which PPARδ attenuates NAFLD remains vague.

To gain insight into the association between PPARδ and NAFLD, we examined whether PPARδ works against the pathogenesis of NAFLD both in vivo and in vitro. We studied the effects of adenovirus-mediated overexpression and agonist induction of PPARδ. We demonstrate that autophagy is associated with PPARδ-induced hepatic fat clearance in vivo by using two rodent models, the db/db mouse and the high fat diet-fed mouse, which have been shown previously to mimic human hepatic steatosis. We also show that PPARδ activation-induced fatty acid oxidation (FAO) mediated by the autophagy–lysosomal pathway is the central mechanism for improving NAFLD.

## Results

### Downregulation of PPARδ and autophagy in the liver of obese mice and ageing mice

**Fig. 1:**
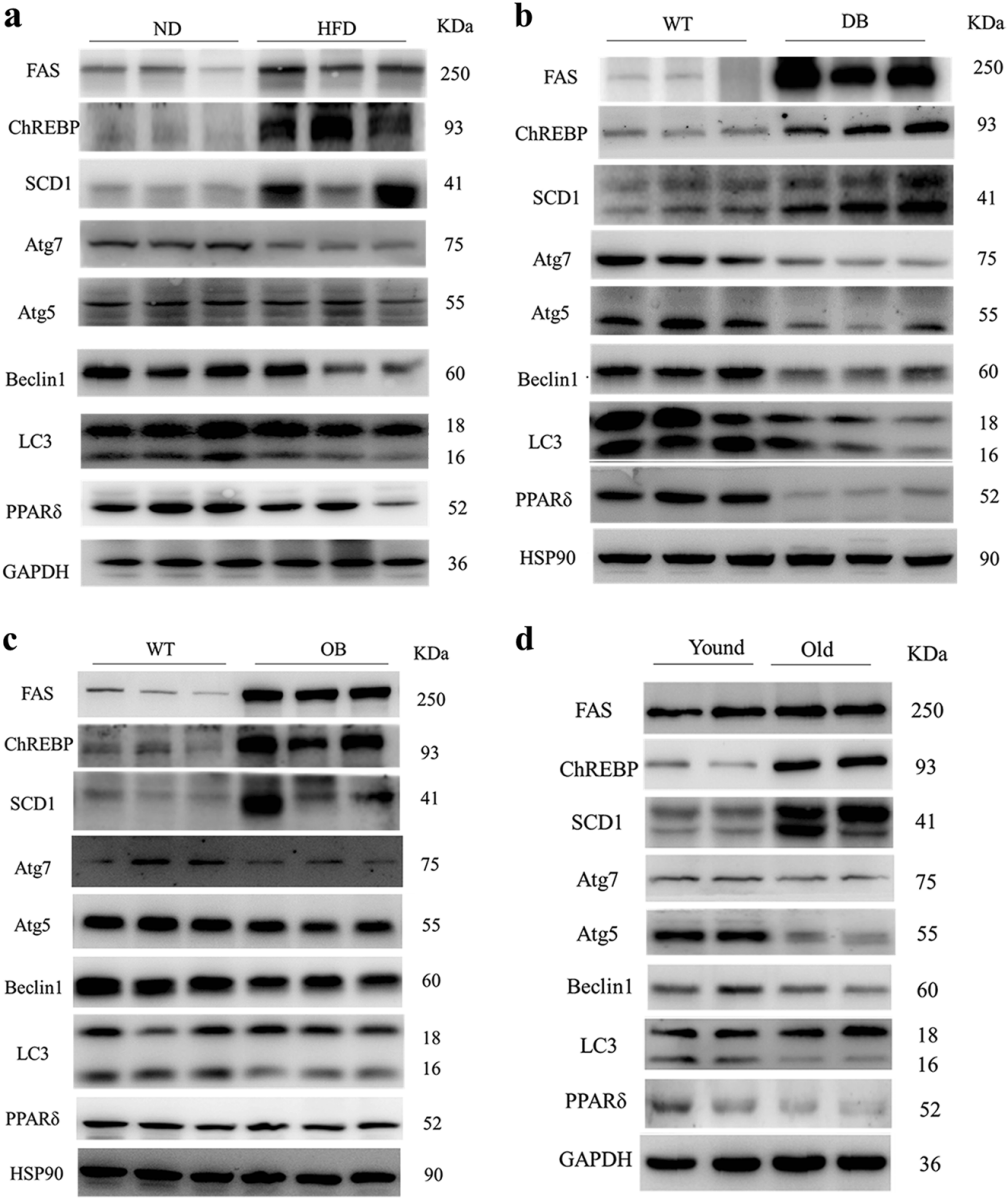
Reduction in PPARδ and autophagy markers in the liver of obese mice. Hepatic protein levels of PPARδ and autophagy markers decreased, accompanied by an increase in genes involved in the de novo lipogenesis pathway, in different obese mouse models: **a** C57BL/6 mice (*n* = 6 per group), **b** db/db mice (*n* = 6 per group) and **c** ob/ob mice (*n* = 6 per group). **d** Effects of ageing on lipid metabolism and autophagy as shown by western blot (*n* = 6 per group)

The most prominent characteristic of NAFLD is abnormal lipid accumulation in the liver. We selected several models of murine obesity, including both dietary (high fat diet) and genetic (ob/ob and db/db) models. The expression of lipogenic proteins, including fatty acid synthase (FAS), carbohydrate-responsive element binding protein (ChREBP) and stearoyl-CoA desaturase 1 (SCD1), was upregulated in model mice compared to control mice (Fig. 1a–c), which is consistent with increased lipid aggregation in the liver of obese mice^19^. PPARδ expression was significantly lower in obese mice than in the respective control mice (Fig. 1a–c). Autophagy proteins showed a significant decrease in obsess mice compared with lean control mice, supported by the downregulation of Atg7, Atg5, Beclin1 and LC3-II (Fig. 1a–c). Normal and pathological ageing is often associated with a reduced autophagic potential. The expression of lipogenic genes was increased in older mice compared to younger mice, and that of autophagic proteins, including Atg7, Atg5, Beclin1 and LC3-II, was significantly decreased. Moreover, PPARδ protein levels were reduced in the ageing mice (Fig. 1d). Altogether, the above results suggest that PPARδ might have some relationship with autophagy.

### PPARδ ligand activation upregulates autophagy in primary hepatic cells

**Fig. 2:**
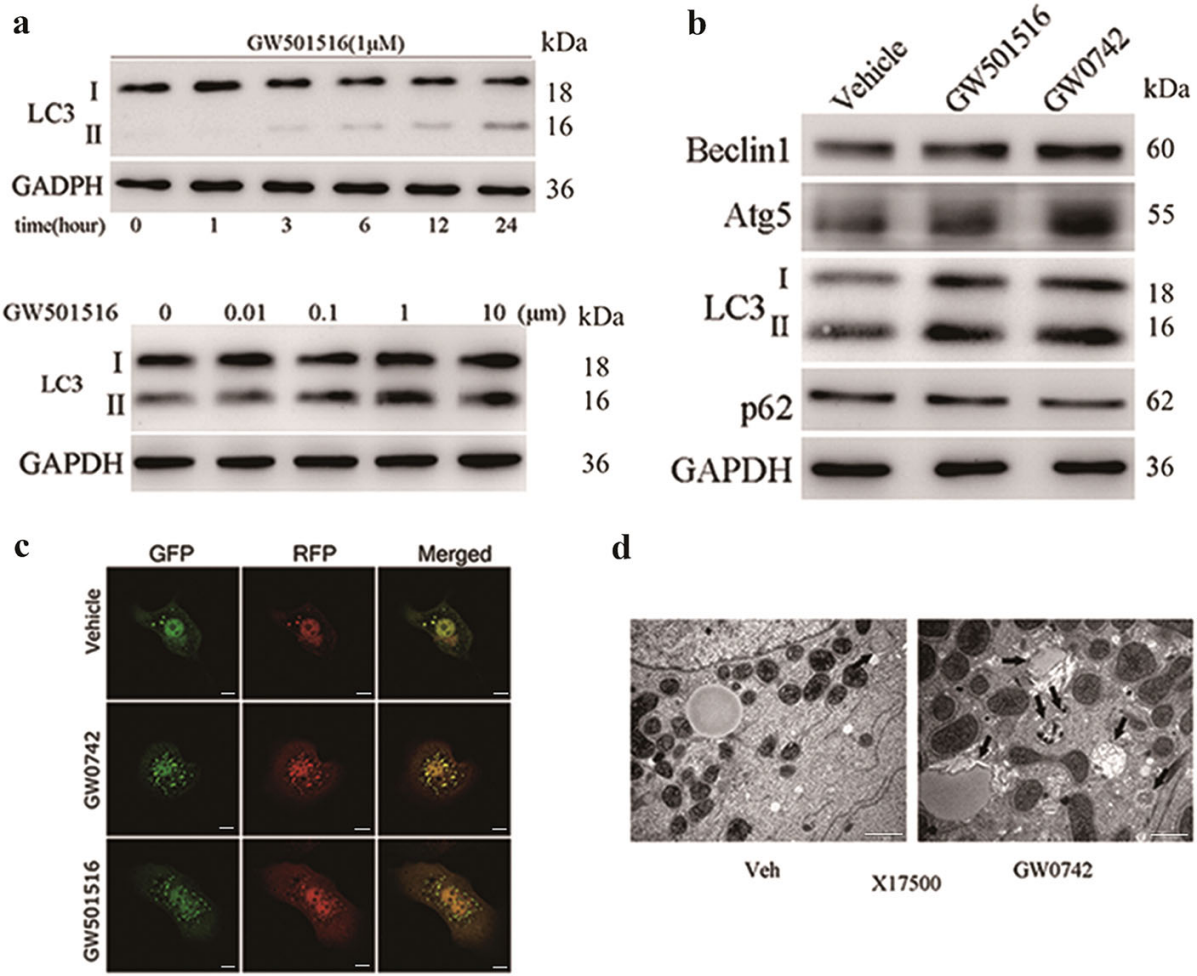
PPARδ ligands induce autophagy in PMH. **a** PMH were treated with the PPARδ ligand GW501516 (1 μM) for the indicated time (0, 1, 3, 6, 12, or 24 h) or at different concentrations (0.01, 0.1, 1 or 10 μM) for 24 h, and LC3-II was examined by western blot. **b** Western blot analysis of autophagy markers after hepatocytes were treated with a PPARδ ligand (1 µM GW0742 or GW501516) for 24 h. **c** Ad-GFP-LC3-infected hepatocytes were treated with GW0742 or GW501516 (both at 1 μM) for 12 h and examined by fluorescence microscopy. Scale bar: 10 μm. **d** Representative EM images of PMH treated with GW0742 for 24 h. Arrows indicate autophagosomes. Scale bar: 1 μm. Three independent experiments were performed

To explore the effect of PPARδ on autophagy, we performed an in vitro study with primary mouse hepatocytes (PMH). Treatment with the PPARδ agonist GW501516 increased LC3-II in PMH in a time- and dose-dependent manner (Fig. 2a upper and lower panels). We then treated PMH with two different PPARδ ligands, which resulted in a marked increase in autophagic proteins, including LC3-II, Atg5 and Beclin1 and the downregulation of p62 (Fig. 2b).

We next used fluorescence microscopy to observe the effect of PPARδ agonists on morphological changes associated with autophagy. Adenovirus carrying the mRFP-GFP-LC3 reporter was used to determine autophagy flux. In this reporter system, mRFP and GFP are used to label and track LC3. While GFP is sensitive to the acidic conditions in autolysosomes, we can detect RFP signals only after the fusion of autophagosomes with lysosomes. The overlay of green and red fluorescence creates yellow dots, and an increase in red spots indicates greater maturation of autolysosomes^20,21^. We observed that PPARδ agonists increased both autophagosome (yellow dots) and autolysosome (remaining red dots) formation in merged images (Fig. 2c). Moreover, PPARδ agonist-treated PMH were evaluated by electron microscopy (EM). Hepatocytes treated with GW0742 had a significantly increased number of autophagic vacuoles compared with those treated with vehicle control (Fig. 2d). Collectively, these results demonstrate strong proautophagic actions upon PPARδ induction.

### PPARδ ligand increases hepatic lipolysis and overall oxidative metabolism in vivo

We conducted some preliminary experiments and generated four sets of mouse models. First, we treated high fat diet (HFD)-fed mice and db/db mice fed a normal chow diet with the PPARδ agonist GW501516. After 4 weeks of GW501516 treatment, fat deposition in liver tissue was significantly improved. Serum TG, ALT and AST levels were decreased, and the mRNA and protein levels of autophagy markers in liver tissue were significantly enhanced (Supplementary Figures S1 and S3). Next, we injected adeno-associated virus (AAV) containing PPARδ or GFP (as a control) through the tail vein into C57BL/6 mice that had been fed a HFD and into db/db mice. For 4 weeks after AAV injection, C57BL/6 mice continued to be fed a HFD, and db/db mice were fed a normal chow diet. Both HFD-fed and db/db mice showed significant improvements in indicators, including serum TG, ALT and AST levels and hepatic TG content. Moreover, autophagy and genes related to FAO were also markedly enhanced compared to control mice (Supplementary Figures S2 and S4).

Chloroquine (CQ) is a well-known pharmacological inhibitor of autophagic and lysosomal function used in vivo^22^ To understand the role of autophagy in PPARδ activation-induced lipid turnover in vivo, we co-administered an autophagy inhibitor to PPARδ agonist-treated genetic obesity models. Obese db/db mice were separated into four groups, each with the same body weight distribution. The four groups were continuously fed vehicle, GW501516 (oral gavage of 10 mg/kg b.w. for 7 days)^23^, CQ (IP administration of 30 mg/kg b.w. for the last 3 days) or GW501516 plus CQ.

**Fig. 3:**
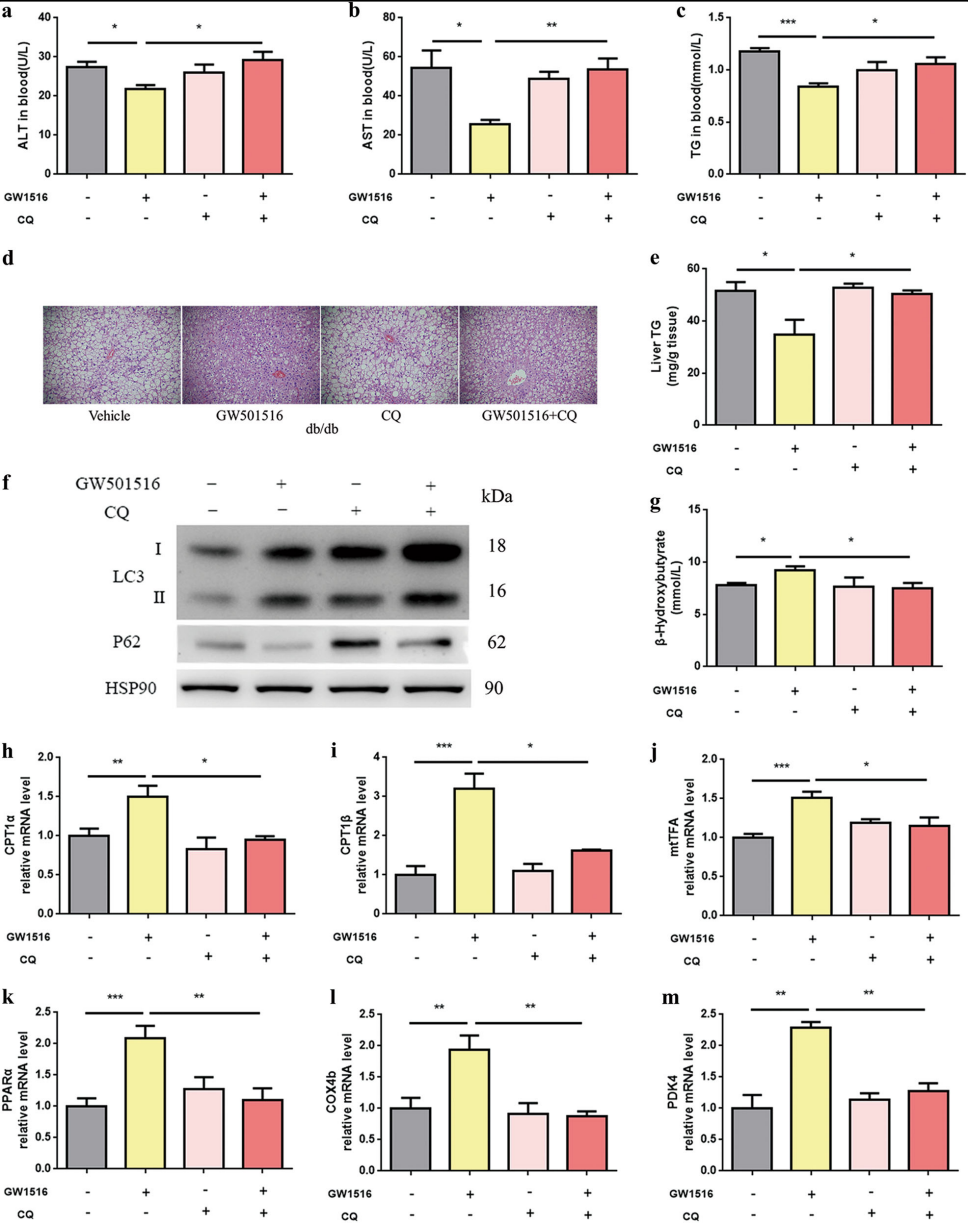
Effects of rapid PPARδ agonist administration on hepatic autophagy and intracellular lipids in db/db mice. **a**–**c** Serum ALT, AST and TGs after 7 days of GW501516 treatment (*n* = 4–6 per group). **d** Histological analysis of H&E-stained liver sections (*n* = 6 per group). Magnification: ×200, scale bar: 50 μm. **e** Hepatic TG content (*n* = 4–6 per group). **f** Western blot (*n* = 4–6 per group). **g** β-OHB levels in serum (*n* = 4–5 per group). **h**–**m** QPCR analysis of genes involved in fatty acid β-oxidation in liver tissue samples (*n* = 5–6 per group). Data are expressed as the mean ± SEM. *P < 0.05, **P < 0.01, ***P < 0.001

After 7 days of treatment, serum ALT and AST levels, as well as serum TGs, were reduced by GW501516. However, in contrast to untreated mice, the GW501516-mediated protective effect on liver was abrogated by CQ administration (Fig. 3a–c). Haematoxylin and eosin (H&E) staining (Fig. 3d) and biochemical analysis of hepatic TG content (Fig. 3e) showed that the steatosis improved by GW501516 treatment was also abolished by CQ administration. Thus, we found that even relatively short-term agonist treatment leads to a vast reduction in lipid accumulation in the liver.

We next sought to explore whether PPARδ activation regulates autophagy flux in the liver. The autophagy-related protein LC3-II was upregulated, and the expression of p62 (an autophagy substrate degraded though autophagy) was reduced in GW501516-treated db/db mice, but this effect disappeared after CQ administration (Fig. 3f). We also performed in vitro study with PMH, PPARδ agonist GW501516 or GW0742 treatment also increased LC3-II in PMH (Supplementary Figure S5).

Autophagy is a catabolic process by which cells supply amino acids and FFAs from self-digested organelles and lipids as an alternative energy source for survival. Therefore, the upregulation of autophagy would eventually be associated with the activation of mitochondrial β-oxidation^21,24^. β-Hydroxybutyrate (β-OHB) is an end product of hepatic FAO. We measured serum β-OHB levels and assessed the mRNA expression of fatty acid β-oxidation-related genes by real-time PCR. GW501516 treatment increased serum β-OHB levels and the mRNA expression levels of CPT1α, CPT1β, mtTFA, PPARA, COX4b and PDK4. However, the GW501516-mediated increases in β-OHB and FAO were abrogated by CQ administration (Fig. 3g, h–m), suggesting that the induction of fatty acid β-oxidation by GW501516 is dependent upon the autophagy–lysosomal pathway.

### The autophagy–lysosomal pathway is essential for the stimulation of β-oxidation by conditional liver PPARδ overexpression in vivo

To establish a direct link between PPARδ-induced autophagy and hepatic fatty acid β-oxidation, db/db mice with conditional liver PPARδ overexpression were treated with or without CQ. The four groups received injections of AAV-GFP (1 × 10^11 vg/ml for 4 weeks), AAV-PPARδ (1 × 10^11 vg/ml), AAV-GFP plus CQ (IP administration of 30 mg/kg for the last 3 days), or AAV-PPARδ plus CQ.

**Fig. 4:**
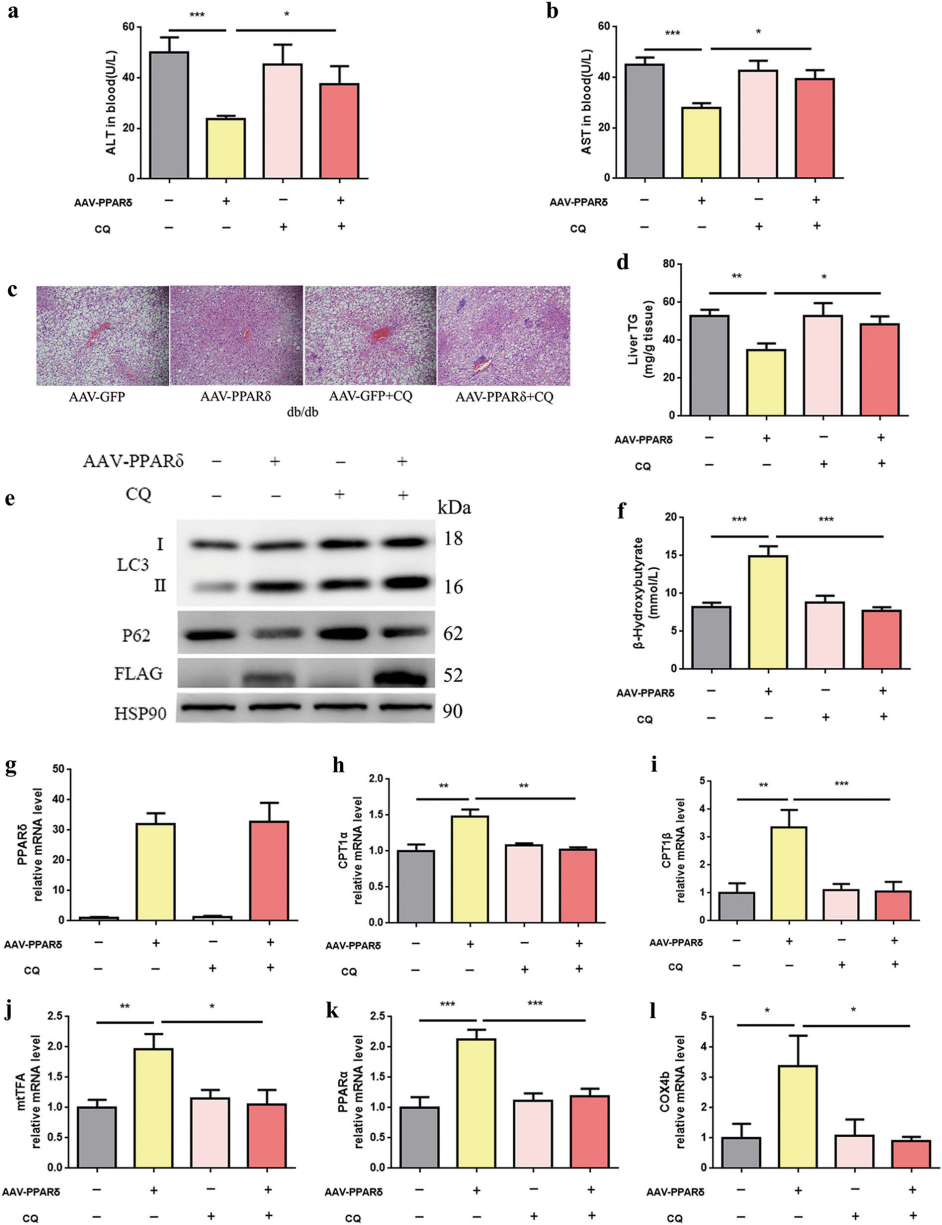
In vivo effects of PPARδ on hepatic autophagy. The db/db mice were intravenously infected with AAV-GFP or AAV-PPARδ for 4 weeks. **a**, **b** Serum ALT and AST four weeks after AAV injection (*n* = 5–6 per group). **c** Liver sections were stained with H&E (*n* = 6 per group), and **d** hepatic TG content was quantified (*n* = 4–6 per group). Magnification: ×200, scale bar: 50 μm. **e** Western blot analysis of LC3, p62, FLAG and HSP90 (*n* = 4–6 per group). **f** β-OHB levels in serum (*n* = 4–5 per group). **g**–**l** QPCR analysis of genes involved in fatty acid β-oxidation in liver tissue samples (*n* = 5–6 per group). Data are expressed as the mean ± SEM. *P < 0.05, **P < 0.01, ***P < 0.001

Similar to the results for obese mice fed GW501516 plus CQ, the reduction in serum ALT and AST levels in the AAV-PPARδ group vanished completely after CQ administration (Fig. 4a, b). Furthermore, H&E-stained liver sections showed that hepatic steatosis was greatly improved, and PPARδ overexpression significantly decreased hepatic TG levels in a quantitative assay. However, these beneficial effects were also abolished by CQ administration (Fig. 4c, d).

The protein levels of autophagy markers, such as LC3, Atg5, Atg7 and Beclin1, increased markedly in the AAV-PPARδ group, and p62 was significantly downregulated. However, the effects disappeared totally after CQ co-administration (Fig. 4e). Conditional PPARδ overexpression increased ketogenesis and the expression of genes related to FAO, and these changes were abrogated in the presence of CQ (Fig. 4f, g–l). These data suggest that PPARδ-induced β-oxidation occurs specifically through the activation of autophagy in vivo.

### Autophagy mediates PPARδ-induced oxidative metabolism in hepatic cells

To determine whether autophagy induced by PPARδ is directly involved in reducing intracellular lipid content, we assessed the effects of lysosomal inhibition on the reduction in intracellular lipids by PPARδ in PMH. We used the PPARδ agonists GW0742 and GW501516 and the autophagy inhibitors CQ and 3-methyladenine (3-MA) in our study. CQ can interfere with the lysosome pH and thus suppress lysosome-mediated degradation, which would lead to the inhibition of autophagy^25^. 3-MA, a widely used autophagy inhibitor, inhibits the class III PI-3 kinase required for autophagy induction, which has been shown to dampen autophagy^20,22^.

**Fig. 5:**
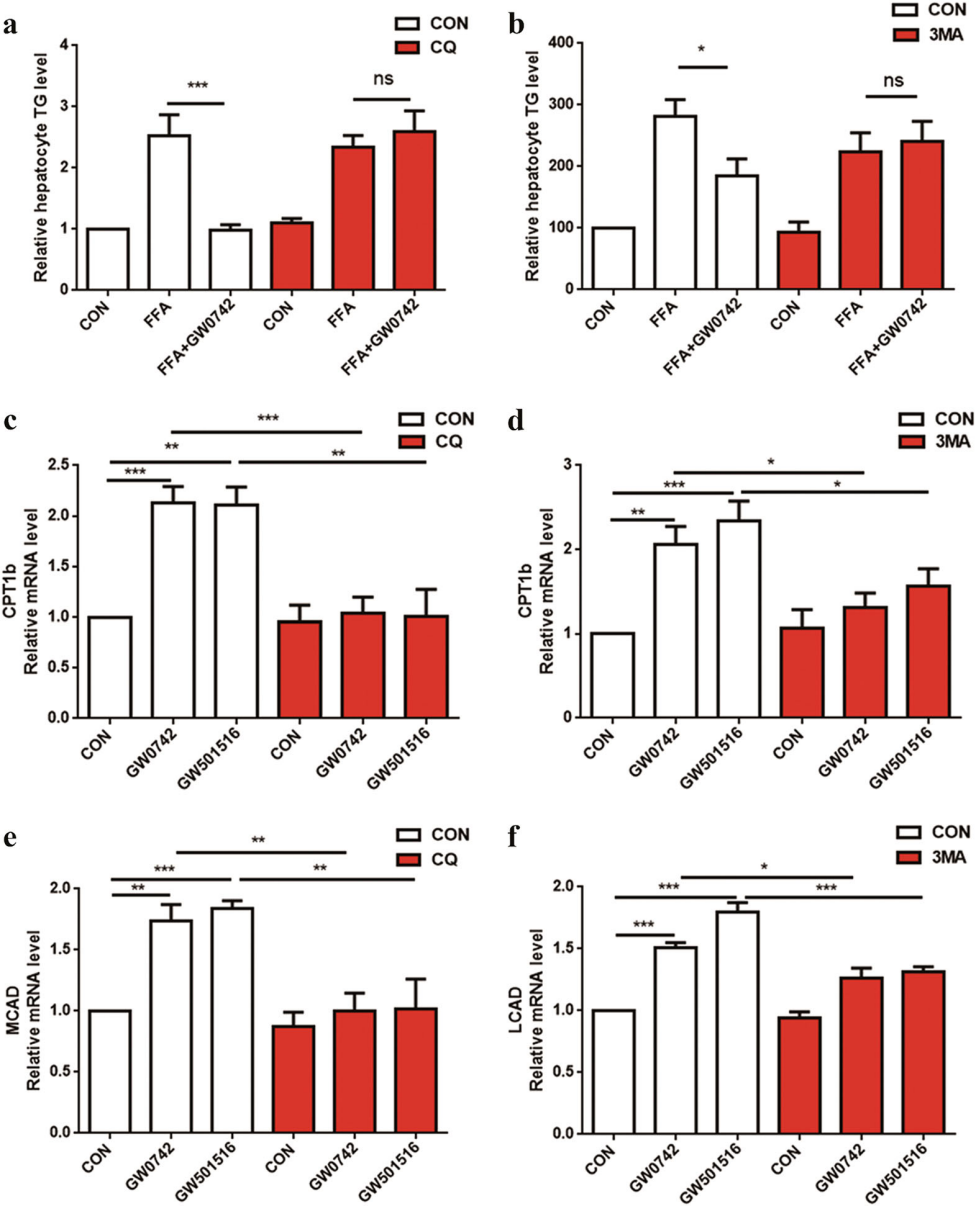
Inhibition of autophagy blocks the PPARδ-mediated reduction of intracellular lipids and FAO in hepatic cells. **a** PMH were cotreated with 1 mM FFAs (OA:PA = 2:1) and the PPARδ ligand GW0742 (1 μM) ± the lysosomal inhibitor CQ (20 μM) for 24 h. TG content was quantified. **b** TG content was quantified with or without 10 mM 3-MA pretreatment for 2 h, and three subgroups were analysed after 24 h: blank, 1 mM FFAs (OA:PA = 2:1), and 1 mM FFAs plus 1 μM GW0742. **c**, **e** PMH were treated with GW0742 (1 μM) or GW501516 (1 μM) for 24 h in the presence or absence of CQ (20 μM), and then, total RNA was extracted for gene expression analysis. **d**, **f** PMH were treated with GW501516 (1 μM) or GW0742 (1 μM) for 24 h in the presence or absence of 3-MA (5 mM), and then, total RNA was extracted for gene expression analysis. Data are expressed as the mean ± SEM of 3 independent experiments. *P < 0.05, **P < 0.01, ***P < 0.001

As shown in Fig. 5a, b, treatment with 1 mM FFAs significantly increased cellular TG levels, and GW0742 significantly weakened the FFA-induced lipid accumulation in PMH. However, this improvement in lipid accumulation upon PPARδ activation in hepatocytes was completely abolished by pretreatment with CQ or 3-MA (Fig. 5a, b). CQ and 3-MA also abolished the increased expression of genes related to fatty acid β-oxidation, which suggested that autophagy is essential for PPARδ-induced fat oxidation and TG clearance (Fig. 5c–f). The above data demonstrate the involvement of the autophagy–lysosomal pathway in the reduction in hepatic intracellular lipids by PPARδ.

### Autophagy inhibition impairs the PPARδ-induced effects on intracellular lipid content and oxidative metabolism in vitro

**Fig. 6:**
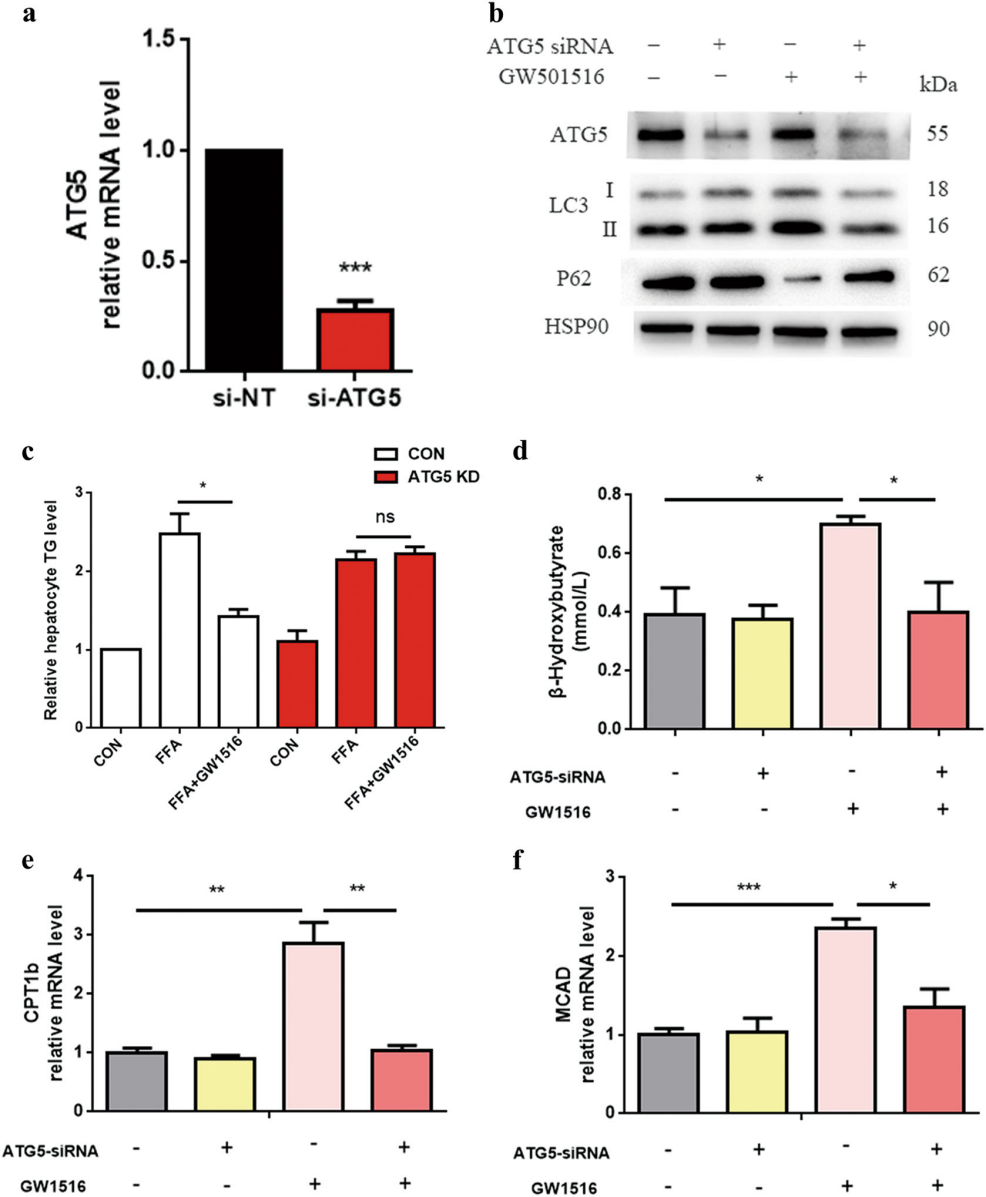
Role of autophagy in the increased oxidative metabolism and reduced intracellular lipids in HepG2 cells. **a** HepG2 cells were transiently transfected with 60 nmol/L ATG5 siRNA or negative control siRNA for 48 h, and then, QPCR analysis of the ATG5 gene was assessed to indicate knockout efficiency. **b** HepG2 cells were first treated with 60 nmol/L ATG5 siRNA for 24 h and then with 1 mM FFAs (OA:PA = 2:1) ± the PPARδ ligand GW501516 (1 μM) for another 24 h. Control cells were fat-loaded cells treated with negative control siRNA with or without GW501516. Western blot analysis of autophagy markers. **c**, **d** TG content and β-OHB measurement in culture media from HepG2 cells ± ATG5 KD (60 nmol/L) for 24 h, followed by a 24-hour treatment with or without 1 mM lipid mixture containing OA and PA at a 2:1 ratio ± GW501516 (1 μM). Control cells were fat-loaded cells treated with negative control siRNA with or without GW501516. **e**, **f** QPCR analysis of genes involved in fatty acid β-oxidation. Data are expressed as the mean ± SEM of 3 independent experiments. *P < 0.05, **P < 0.01, ***P < 0.001

We used ATG5 siRNA to block autophagosome formation. The data showed that ATG5-targeted RNA interference efficiently abrogated ATG5 gene and protein expression (Fig. 6a, b). ATG5 knockdown (KD) impaired PPARδ agonist-induced effects. Additionally, ATG5 KD abolished GW501516-induced autophagy by decreasing the expression of the autophagy-related protein LC3-II and increasing the protein expression p62, an autophagy substrate (Fig. 6b).

GW501516 treatment increased the expression of genes related to FAO, but this effect was blocked by ATG5 KD (Fig. 6e, f). Furthermore, ATG5 KD also abolished PPARδ activation-induced β-OHB production in HepG2 cells (Fig. 6d). These results suggest that autophagy is essential for PPARδ-induced FAO.

Most importantly, ATG5 siRNA dramatically impaired the downregulation of intracellular lipids by GW501516, strongly suggesting the involvement of autophagy in the PPARδ-induced reduction of intracellular lipids (Fig. 6c).

### PPARδ is required to upregulate autophagy in vitro

**Fig. 7:**
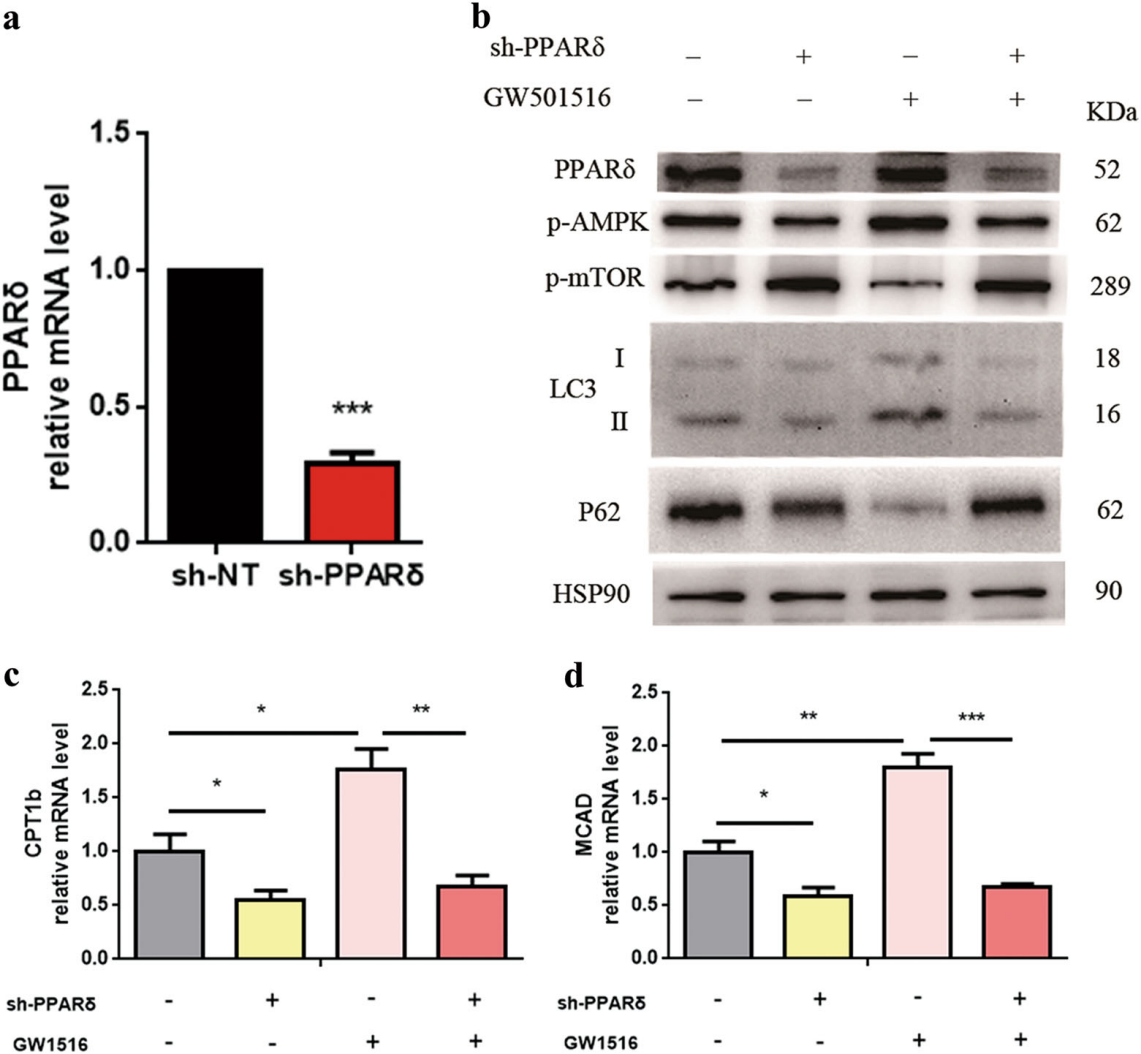
PPARδ is required to upregulate autophagy in vitro. **a** HepG2 cells were infected with lentivirus-mediated control shRNA or PPARδ shRNA at a multiplicity of infection of 10 in 6-well plates for 72 h; then, QPCR analysis of PPARδ gene expression was performed to assess the knockout efficiency. **b** HepG2 cells were first infected with lentivirus-mediated control shRNA or PPARδ shRNA for 24 h and then treated with the PPARδ ligand GW501516 (1 μM) for another 72 h. Control cells were cells treated with negative control shRNA with or without GW501516. Protein expression levels were determined by western blot. **c**, **d** QPCR analysis of genes involved in fatty acid β-oxidation. Data are expressed as the mean ± SEM of 3 independent experiments. *P < 0.05, **P < 0.01, ***P < 0.001

The short hairpin RNA (shRNA)-mediated KD of PPARδ was used to assess the specific function of PPARδ in the regulation of autophagy in vitro. As shown in Fig. 7a, b, PPARδ-targeted RNA interference efficiently abrogated PPARδ gene and protein expression. We found that PPARδ KD was sufficient to inhibit autophagy under basal conditions, as reflected by decreased LC3-II levels and increased p62 expression. Furthermore, PPARδ KD significantly decreased the phosphorylation of AMP-activated protein kinase (AMPK) and increased p-mammalian target of rapamycin (mTOR) levels (Fig. 7b). PPARδ KD also abolished GW501516-induced FAO in HepG2 cells (Fig. 7c).

### PPARδ facilitates autophagy through AMPK/mTOR pathway

**Fig. 8:**
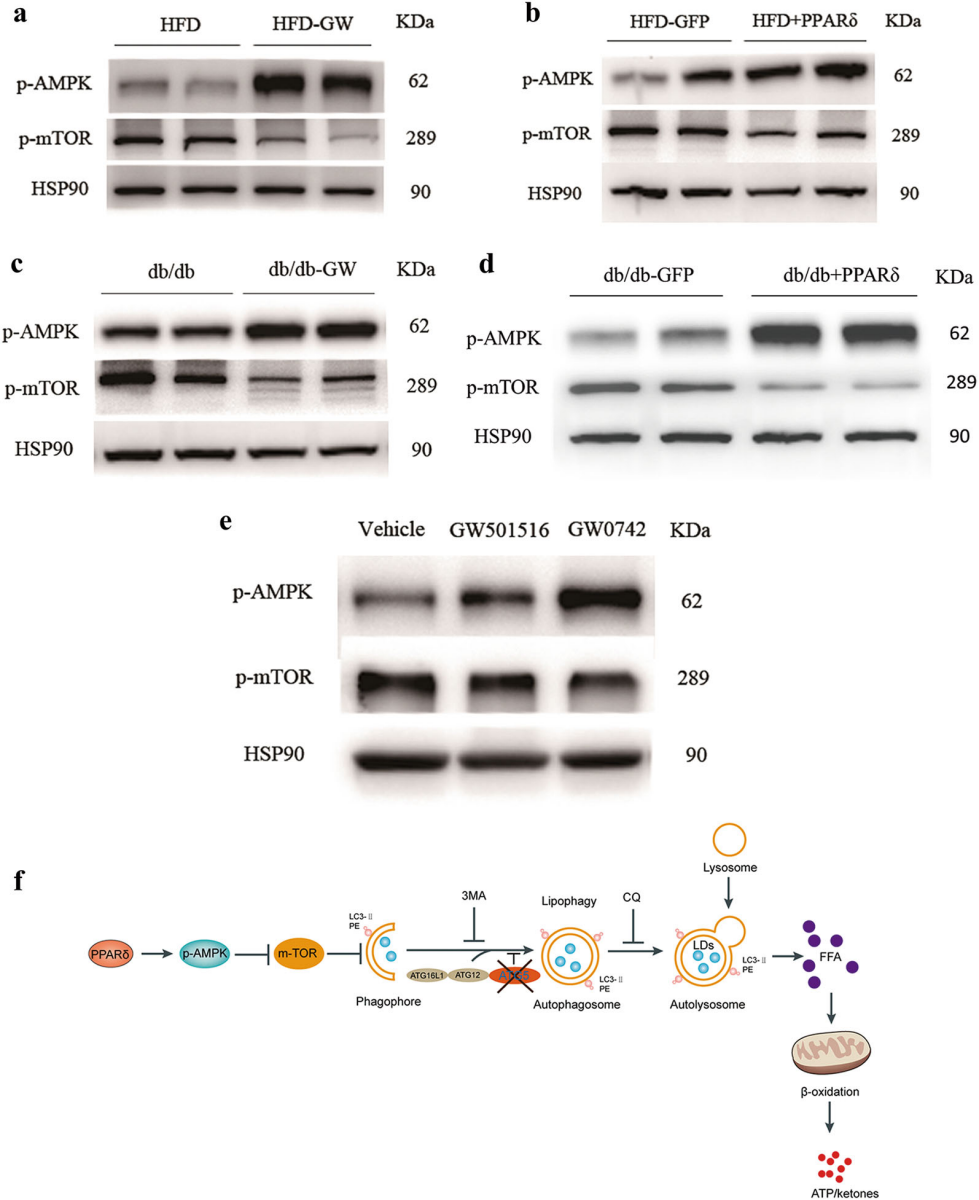
The induction of PPARδ-induced FAO mediated by autophagy-lysosomes through AMPK/mTOR pathway. The phosphorylation levels of AMPK and mTOR in vivo and in vitro. **a** HFD and HFD + GW501516 mice (*n* = 4–6 per group). **b** HFD + GFP and HFD + PPARδ mice (*n* = 4–6 per group). **c** db/db and db/db + GW501516 mice (*n* = 4–6 per group). **d** db/db + GFP and db/db + PPARδ mice (*n* = 4–6 per group). **e** PMH were treated with the PPARδ ligand GW501516 (1 μM) or GW0742 (1 μM) for 24 h. Three independent experiments were performed. **f** PPARδ can increase the phosphorylation of AMPK, and the nutrient-sensing kinase mTOR is inhibited by AMPK. Autophagy is known to be promoted by AMP, so the inhibition of mTOR may activate autophagy through the PPARδ/AMPK/mTOR pathway. Autophagosomes fuse with lysosomes to form mature autolysosomes, in which engulfed contents, including cytosolic lipid droplets (LDs), are degraded. After autolysosome formation, the breakdown of TGs in LDs generates FFAs, which can undergo β-oxidation for ATP and ketone production. PPARδ can activate autophagy, thus increasing LD degradation and hepatic FAO. Two widely used autophagy inhibitors, 3-MA and CQ, and ATG5 KD can also block the effects of PPARδ. The diagram shows that PPARδ has a beneficial effect in the reduction in intrahepatic lipid content and stimulates β-oxidation by an autophagy–lysosomal pathway

We measured the phosphorylation levels of AMPK and mTOR levels in vivo, and in all four different mouse models, we found that AMPK phosphorylation was significantly increased and that mTOR phosphorylation was decreased by PPARδ overexpression (Fig. 8a–d). Next, we performed an in vitro study with PMH and treated these cells with two different PPARδ ligands, GW501516 and GW0742. The results showed a marked increase in p-AMPK and the downregulation of p-mTOR (Fig. 8e).

A schematic diagram of the induction of PPARδ-induced FAO mediated by the autophagy–lysosomal pathway is shown in Fig. 8f.

## Discussion

In this study, we have shown that PPARδ overexpression or agonist induction prevents hepatic steatosis in obese mice. Our findings are concordant with those of a previous clinical trial, which showed a reduction in liver fat in moderately obese men after two weeks of GW501516 treatment^18^. Here, we also identified and characterised a novel mechanism by which PPARδ effectively increases hepatic FAO and autophagic flux. Our study may provide a critical mechanism to narrow the gap between basic research and clinical applications.

Although increased supply of FFAs, hepatic lipogenesis, decreased FAO, and the hepatic secretion of TG-rich, very low-density lipoprotein have all been observed in the development of NAFLD, the primary reason for lipid accumulation in the liver is not yet clear^26–28^. In recent years, there has been intense interest in autophagy, which could be manipulated for the management of NAFLD^26,29^. As reported in 2009 by Rajat Singh et al., the inhibition of autophagy in cultured hepatocytes and the mouse liver increased TG storage in lipid droplets^30^, and autophagy-enhancing drugs could alleviate liver steatosis in HFD-fed mice^31^. It should be noted that some common drugs used in the treatment of metabolic disease, such as metformin, mitigate hepatic fat content in a manner involving the autophagy machinery^32^. In particular, we showed that PPARδ effectively decreases intracellular TGs and increases autophagic flux in primary hepatic cells and in animals. Activation of the autophagy machinery was demonstrated on several levels, including by examining LC3 turnover in the absence or presence of CQ to measure the induction of autophagy and by using fluorescence microscopy to visualise autophagosome formation via LC3 localisation. Although the experiments were performed in different tissues, other researchers have demonstrated that GW501516 induces autophagic markers in cultured cardiac cells and in the heart of HFD-fed mice^33,34^.

In fasting conditions, the body can hydrolyse TG to supply FFAs that meet the energy needs^35^. Hepatic FAO is enhanced in the fasted state^36^ and an alternative energy source with respect to energy deprivation is provided by the breakdown of cellular components by autophagy^35,37^. Defective autophagy is involved in NAFLD^30^, and the induction of autophagy can correct the over-accumulation of lipids in the liver^38^. We used pharmacological (CQ and 3-MA) and genetic (ATG5 siRNA) methods to modulate autophagy and lysosomes to demonstrate that the autophagy–lysosomal pathway is essential for the reduction of intracellular fat and the stimulation of oxidative metabolism by PPARδ. We treated primary hepatic cells with PPARδ in the presence of CQ, 3-MA or ATG5 siRNA to establish a direct link between PPARδ-induced autophagy and hepatic fatty acid β-oxidation. It has been reported that PPARδ can improve hepatic steatosis by activating fatty acid β-oxidation in different diet-induced models of steatohepatitis (NASH, obesity and insulin resistance)^39–41^. Our study clearly demonstrates the importance of autophagy and lysosomal degradation in promoting fatty acid β-oxidation, which might be the key pathway through which PPARδ maintains intrahepatic lipid homoeostasis.

Our results also showed increased AMPK phosphorylation and mTOR inhibition in vivo and in vitro upon PPARδ induction. Autophagy can be promoted by the energy sensor AMP-activated protein kinase (AMPK) and be inhibited by the mammalian target of rapamycin (mTOR)^42^. Although further investigations need to be done, it is possible that the underlying mechanism of PPARδ activated autophagy formation at least partly involves the AMPK/mTOR pathway.

Another reason why PPARδ is a promising drug for targeting NAFLD is its expression in different cell types of the liver. PPARδ is expressed in hepatocytes, Kupffer cells and stellate cells^43^. In accordance with the beneficial effects of PPARδ in improving hepatic steatosis, PPARδ could also protect against liver inflammation and fibrosis^40,43,44^.

In summary, we have demonstrated that PPARδ has a beneficial effect in attenuating hepatic steatosis by activating autophagy in vivo and in vitro. The mobilisation and hydrolysis of TGs to FFAs through the autophagy–lysosomal pathway induced by PPARδ led to increased delivery of FFAs to mitochondria. This, in turn, increased the β-oxidation of fatty acids. These findings are especially noteworthy because it is possible that PPARδ activation will be a pharmacological tool for activating autophagy. Understanding the mechanistic basis of PPARδ provides further insight into the development of more drugs for the prevention and treatment of NAFLD.

## Materials and methods

### Chemicals and antibodies

GW501516, GW0742 and 3-MA were purchased from Sigma-Aldrich (MO, USA). The antibodies used for western blot included anti-LC3A/B, anti-p62, anti-Atg7, and anti-Beclin1 (Cell Signaling Technology); anti-Atg5 (Novus Biologicals); anti-GAPDH (KangCheng Bio-tech); and anti-PPARδ (Santa Cruz Biotechnology). All the other chemicals were from Sigma-Aldrich Corporation unless specifically noted.

### Cell culture

PMH were fractionated by collagenase digestion from wild-type C57BL/6J mice aged 6–10 weeks. After being filtered and washed with PBS, the hepatocytes were seeded on new plates in attachment media (Hepatocyte Medium, ScienCell) for 24 h before further experiments. The culture medium was replaced with DMEM (Dulbecco’s Modified Eagle’s Medium, GIBCO) when the hepatocytes were treated with GW501516 or other chemicals. The cells were always cultured in a 37 °C incubator with 5% CO_2_.

HepG2 cells were maintained at 37 °C in DMEM (GIBCO) supplemented with 10% foetal bovine serum.

### Animal experiments

The animal protocol was reviewed and approved by the Animal Care Committee of Shanghai Jiao Tong University School of Medicine. Male C57BL/6J mice were purchased from Shanghai Laboratory Animal Co., Ltd. (SLAC). Obese C57BLKS/J-Leprdb/Leprdb (db/db) male mice and their wild-type littermates were purchased from the Model Animal Research Center of Nanjing University. At 8 weeks of age, the mice were housed at 22 ± 2 °C and 55 ± 5% relative humidity with a 12-hour light/dark cycle. HFD-induced obese mice had free access to high fat chow (D12492, Research Diets, New Brunswick, New Jersey, USA) and drinking water beginning at 8 weeks old. This diet contains 60% fat, 20% protein and 20% carbohydrate by energy.

### Tandem mRFP-GFP fluorescence microscopy

Adenovirus expressing tandem mRFP-GFP-LC3 was purchased from Hanbio Biotechnology Co., Ltd., and was used to monitor autophagic flux in primary hepatocytes. Twenty-four hours after transfection, RFP and GFP were detected in hepatocytes. Then, GW0742, GW501516 or vehicle was added, and 9 h later, the hepatocytes were washed with PBS and fixed with paraformaldehyde. The samples were sent out to be scanned and photographed by confocal microscopy.

### Transmission electron microscopy

Cells were washed with PBS and then fixed in 2.5% glutaraldehyde in PBS for 2 h at 4 °C. After two washes with PBS, the cells were postfixed in 1% osmium tetroxide in 0.1 M phosphate buffer for 2 h. Samples were dehydrated with increasing concentrations of ethanol and then embedded in epoxy resin. Slices were cut with an ultramicrotome and sent to be photomicrographed under a transmission electron microscope (PHILIP, CM-120, Holland).

### Immunoblotting

Homogenised tissues and cells were lysed in RIPA buffer with protease and phosphatase inhibitors. After separation by SDS-PAGE, the proteins in each sample was transferred to polyvinylidene fluoride membranes (Millipore). The membranes were blocked, incubated with different primary antibodies and then incubated with horseradish peroxidase-conjugated secondary antibodies following the manufacturer’s protocol. Protein bands were visualised by chemiluminescence with ECL Prime Western Blotting Detection Reagent (GE Healthcare) and quantified using AlphaView Software (for results, see Supplementary Fig 6).

### Quantitative real-time PCR analysis

After 1 µg of total RNA was extracted from each sample with Trizol reagent (Invitrogen) following the manufacturer’s instructions, cDNA was prepared using the Reverse Transcription System (Promega). The relative mRNA expression level was determined by quantitative real-time PCR with SYBR Premix (Takara Bio, RR420A) and a Thermo Fisher QS DX Real-time PCR system.

### Histological examination

Liver tissues were fixed in 4% paraformaldehyde overnight immediately after dissection, embedded in paraffin and sectioned. After H&E staining, the slices were visualized and imaged by light microscopy at ×200 magnification.

### Hepatic and cellular TG measurement

Mice were sacrificed after an overnight fast (16 h), and liver tissues were preserved in liquid nitrogen. Approximately 50 mg of liver tissue was sliced and homogenised in 1 ml of 5% NP-40 buffer. Then, the samples were incubated in a water bath at 80–100 °C for 5 minutes, cooled to room temperature; the heating and cooling process was repeated once. Next, the samples were centrifuged at 13,300 rpm for 2 minutes to remove any insoluble material. The TG concentration was measured using a Triglyceride Quantification Kit (Biovison, K622–100). PMH were resuspended in 5% NP-40 buffer, and TG content was detected as described above.

### Autophagy inhibition and intracellular TG measurement in vitro

PMH and HepG2 cells were cotreated with 1 mM FFAs (OA:PA = 2:1) and PPARδ ligand (1 µM GW0742 or GW501516) with or without the autophagy inhibitor CQ (20 μm) or 3-MA (5 mM) for 24 h. TG content was quantified.

### Acute GW501516 and CQ administration and ketogenesis measurements

GW501516 (10 mg/kg b.w.) was orally gavaged daily for 7 days in male db/db mice fed a normal chow diet (NCD), and tissues were collected for western blotting and real-time PCR analysis. To assess the effect of autophagy inhibition on PPARδ-induced fat oxidation in vivo, CQ was injected IP daily for the last 3 days in male db/db mice (30 mg/kg b.w.), and β-OHB levels were assessed using a commercially available colorimetric kit (Jiancheng Bioengineering Institute, Nanjing).

### Liver-restricted PPARδ overexpression and CQ administration in vivo

AAV expressing murine PPARδ was constructed by Hanbio Biotechnology Co., Ltd., with the full-length cDNA for PPARδ containing a FLAG tag. Overexpression of PPARδ was achieved by AAV-PPARδ (1 × 10^11 vg/ml) injection into db/db mice through the tail vein, and AAV-GFP was used as a control. The db/db mice continued to be fed a NCD for another fourweeks after the AAV injection, and CQ (30 mg/kg b.w. IP for the last three days) was co-administered with AAV injections. Tissues were collected for western blotting, real-time PCR analysis and β-OHB measurements.

### Targeted KD of ATG5 by siRNA

ATG5 KD was carried out using Stealth siRNA duplex oligoribonucleotides targeting human ATG5 (Invitrogen). After 24 h, HepG2 cells were treated with the PPARδ ligand GW501516 (1 μM) and 1 mM FFAs (OA:PA = 2:1). Measurements of intracellular levels were assessed after 24 h of cotreatment. The siRNA oligonucleotide duplex (5-GGU UUG GAC GAA UUC CAA CUU GUU U-3) to human ATG5 was synthesised by Invitrogen, Carlsbad, CA (Stealth Select RNAi). Stealth RNAi negative control with low GC content was ordered directly from Invitrogen (catalogue number 12935200). Transient transfection of synthetic siRNA was achieved using Lipofectamine 2000 transfection reagent (Invitrogen). The transfection efficiency of each duplex siRNA (>90%) was confirmed by using BLOCK-iT™ Alexa Fluor™ Red Fluorescent Control (Invitrogen, catalogue number 14750100) according to the manufacturer’s instructions.

### Lentivirus transduction

The lentivirus containing sense–loop–antisense shRNA targeting human PPARδ was purchased from ShanghaiXitubio Corporation. The shRNA sequences were as follows: PPARδ, GATCAAGAAGACCGAAACCGA; and negative control, GCGCGATAGCGCTAATAATTT. PPARδ shRNA was subcloned into the pLKO5-GFP lentiviral vector to obtain the pLKO5-GFP-sh-PPARδ construct. Then, HepG2 cells were infected with pLKO5-GFP-sh-PPARδ or negative lentiviral vectors at a multiplicity of infection of 10. The efficiency of infection at 72 h, which was determined by the number of GFP-positive cells, exceeded 90% for all constructs.

### Statistical analysis

Statistical analyses were performed with SPSS version 23.0 (SPSS Inc., Chicago, IL, USA) and GraphPad Prism version 6.0 (GraphPad Software Inc., San Diego, CA, USA). Continuous data are presented as the mean ± standard error of the mean (SEM) unless otherwise specified. The Kolmogorov–Smirnov test (using SPSS) or Bartlett’s test (using GraphPad Prism) was performed to verify the normality of each variable, and the Brown–Forsythe test was performed to test the equality of group variances. For the comparison of two groups, the unpaired Student’s *t*-test was used if the variances were equal; otherwise, the *t*-test was performed. For the comparison of three or more groups, one-way analysis of variance (ANOVA) with Tukey’s post hoc adjustment was performed. A two-tailed *P* value less than 0.05 was considered statistically significant.

All the data are presented as the mean ± SEM. Statistical differences were set as *p < 0.05, **p < 0.01 and ***p < 0.001.

## Supplementary information

SUPPLEMENTAL MATERIAL
