# Supplementary material for: PPARδ attenuates hepatic steatosis through autophagy-mediated fatty acid oxidation
Source: Cell Death Dis. 2019 Feb 27;10(3):197. doi: 10.1038/s41419-019-1458-8 (PMC6393554; doi:10.1038/s41419-019-1458-8)
Supplement: Supplementary file 1 — SUPPLEMENTAL MATERIAL [file 41419_2019_1458_MOESM1_ESM.docx]

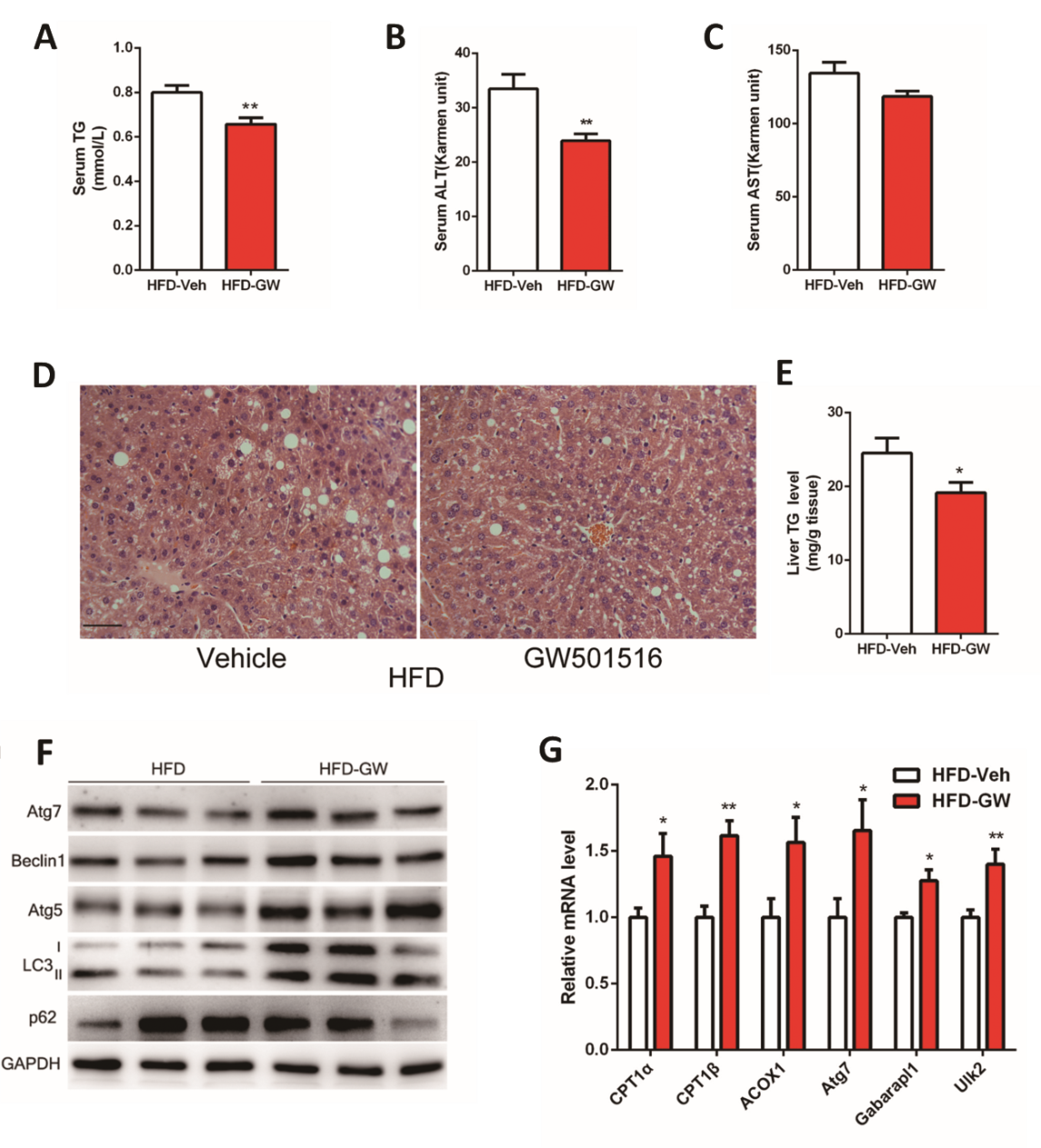


**Supplementary Figure 1 PPARδ agonist ameliorates hepatic steatosis and induces autophagy in HFD-fed mice.** (A-C) Serum TG, ALT and AST levels after four weeks of GW501516 treatment (n = 4-6 per group). (D) Histological analysis of H&E-stained liver sections (n = 6 per group). Magnification: 400×, scale bar: 50 μm. (E) Hepatic TG content (n = 4-6 per group). (F) Liver protein (n = 4-5 per group) and (G) mRNA expression of genes involved in autophagy and fatty acid β oxidation (n = 5-6 per group). Data are expressed as the mean ± SEM. *P < 0.05, **P < 0.01, ***P < 0.001.

**
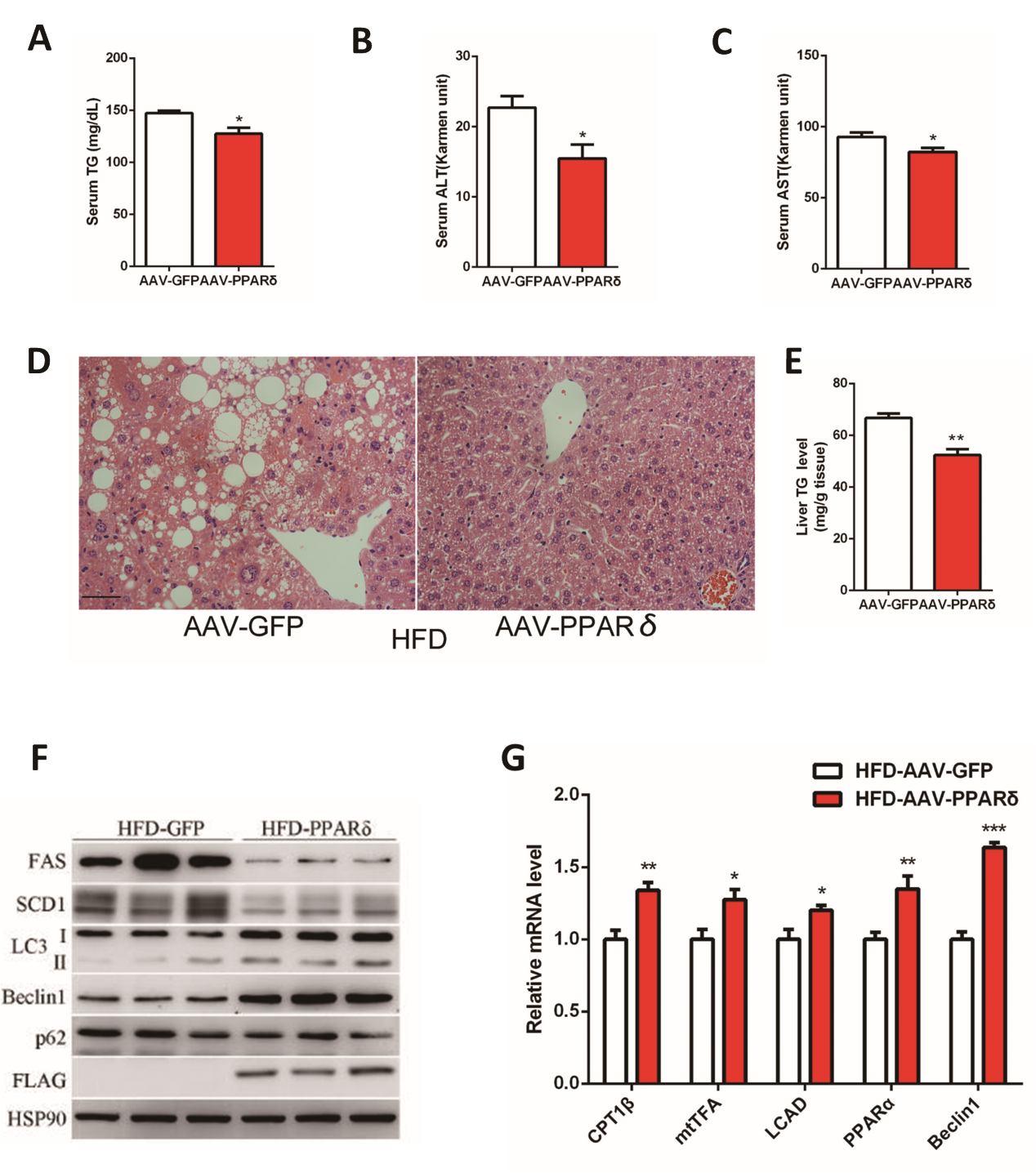
**

**Supplementary Figure 2 Overexpression of PPARδ enhances autophagy flux and reduces hepatic lipid content in HFD-fed mice.** Male HFD mice were intravenously infected with AAV-GFP or AAV-PPARδ for 4 weeks. (A-C) Serum TG, ALT and AST levels four weeks after AAV injection (n = 5-6 per group). (D) Liver sections were stained with H&E (n = 6 per group), and (E) hepatic lipid content was quantified (n = 6 per group). (F) Protein was extracted from liver tissues for western blot experiments using antibodies against FAS, LC3, Beclin1, p62, FLAG and HSP90 (n = 4-5 per group). (G) Changes in the relative mRNA expression of genes involved in fatty acid β-oxidation and autophagy after 4 weeks of PPARδ overexpression(n = 5-6 per group). Data are expressed as the mean ± SEM. *P < 0.05, **P < 0.01, ***P < 0.001.


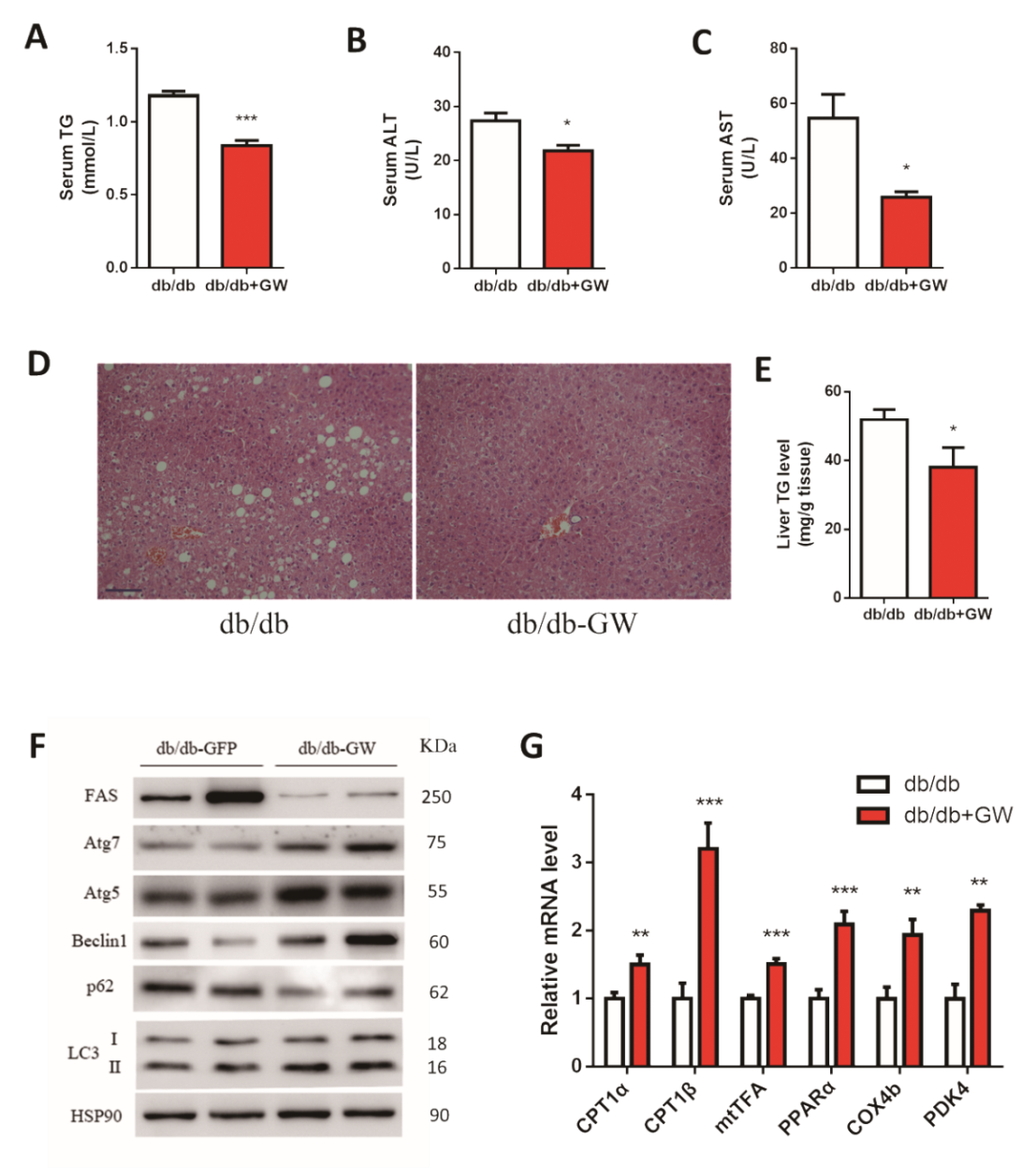


**Supplementary Figure 3 PPARδ agonist ameliorates hepatic steatosis and induces autophagy in db/db mice.** (A-C) Serum TG, ALT and AST levels after four weeks of GW501516 treatment (n = 4-5 per group). (D) Histological analysis of H&E-stained liver sections (n = 6 per group). Magnification: 400×, scale bar: 50 μm. (E) Hepatic TG content (n = 6 per group). (F) Liver protein (n = 4-5 per group) and (G) mRNA expression of genes involved in autophagy and fatty acid β oxidation (n = 4-6 per group). Data are expressed as the mean ± SEM. *P < 0.05, **P < 0.01, ***P < 0.001.

**
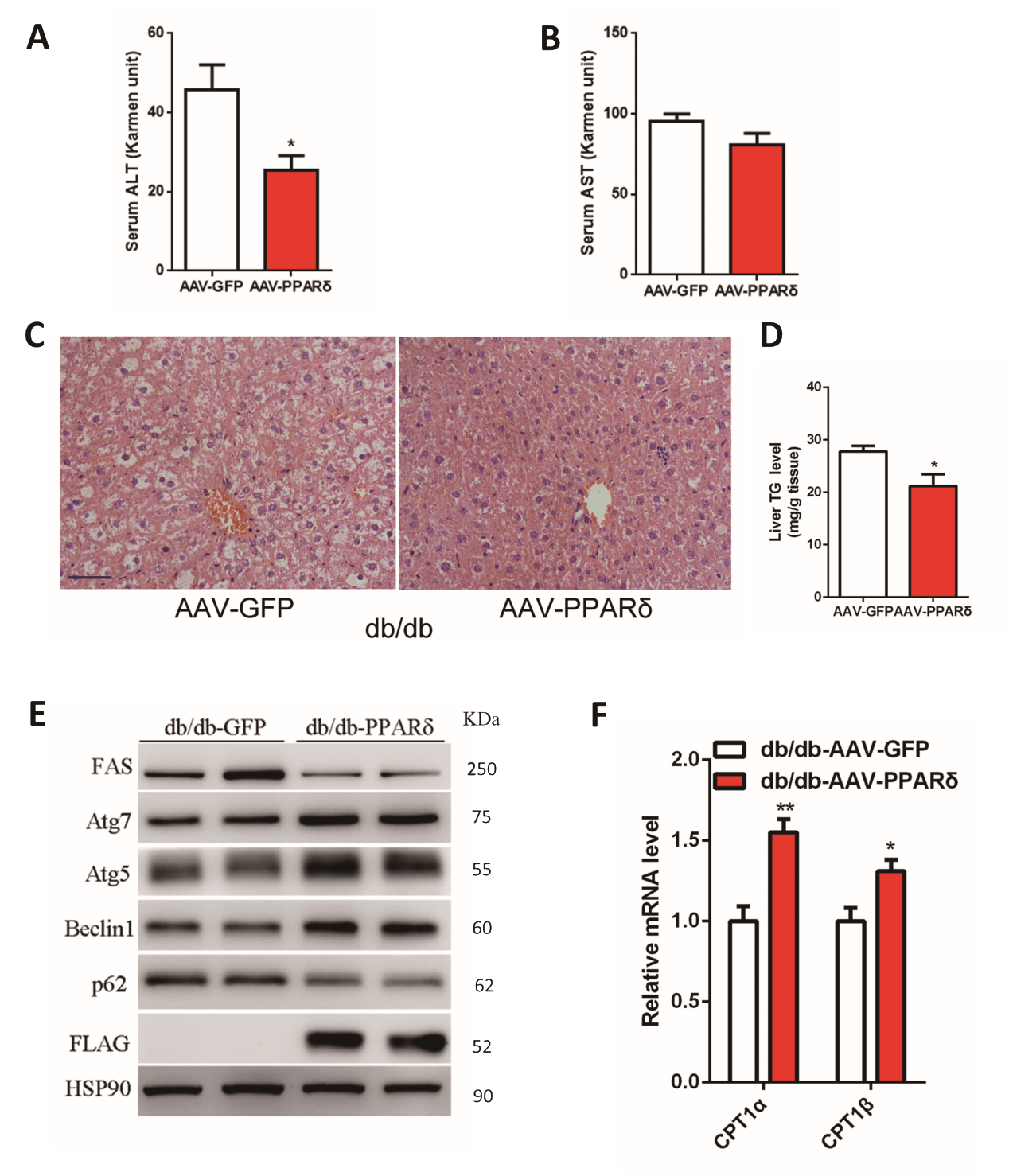
Supplementary Figure 4 Overexpression of PPARδ induces autophagy and improves hepatic steatosis in db/db mice.** Selective overexpression of PPARδ in the liver was achieved via AAV injection of eight-week-old male db/db mice for 4 weeks. (A-B) Serum ALT and AST levels (n = 4-6 per group). (C) Liver sections were stained with H&E (n = 6 per group), and (D) hepatic lipid content was quantified (n = 5-6 per group). (E) Immunoblotting was performed using antibodies against FAS, Atg7, Atg5, Beclin1, p62, FLAG and HSP90 (n = 4-5 per group). (F) The expression of genes related to fatty acid β oxidation was examined using real-time qPCR (n = 5-6 per group). Data are expressed as the mean ± SEM. *P < 0.05, **P < 0.01, ***P < 0.001.


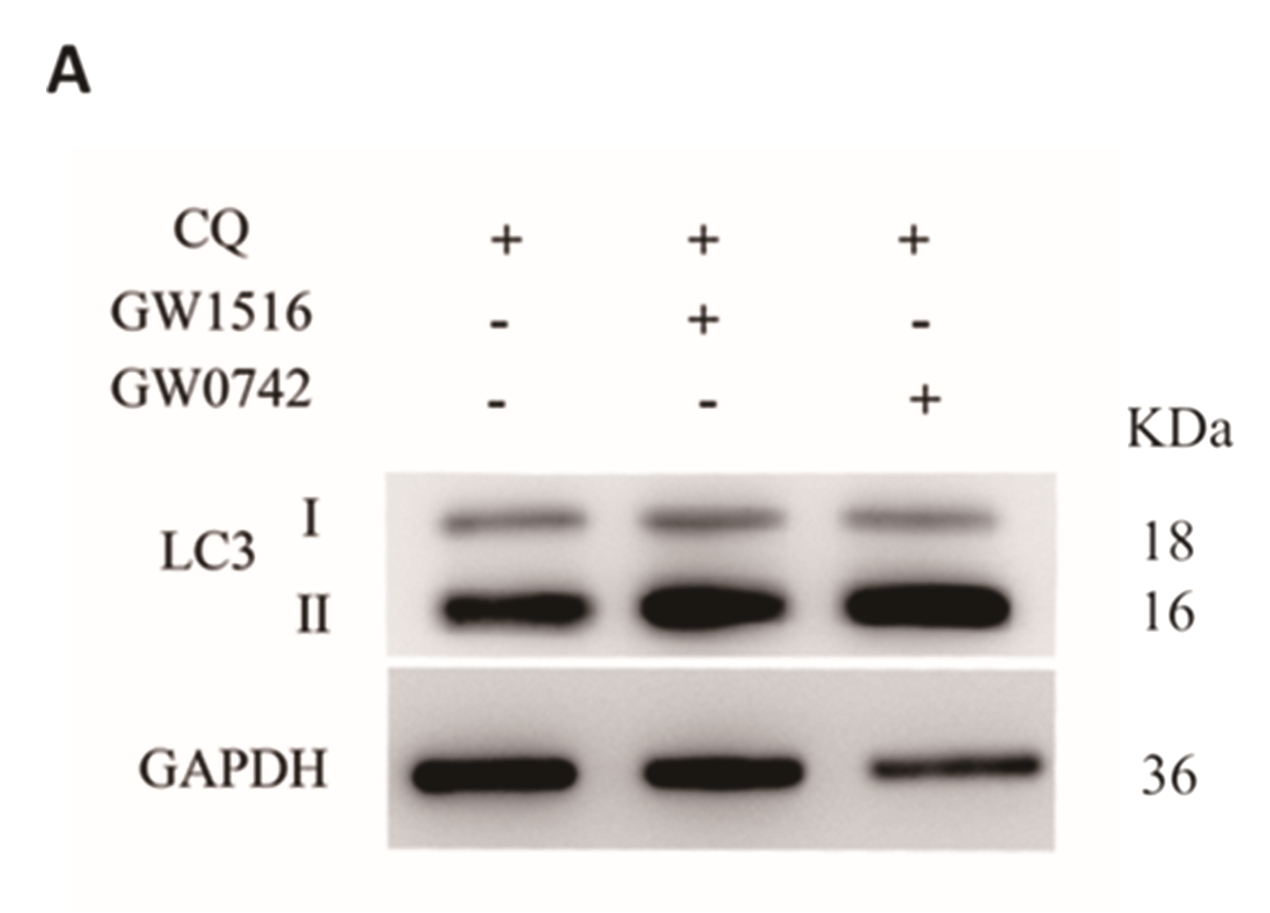


**Supplementary Figure 5** **Primary mouse hepatocytes were treated with DMSO, GW501516 (1 μM) or GW0742 (1 μM) for 24 hours in the presence of CQ (20 μM).** (A) Immunoblotting was performed using an antibody against LC3. Data are expressed as the mean ± SEM of 3 independent experiments.


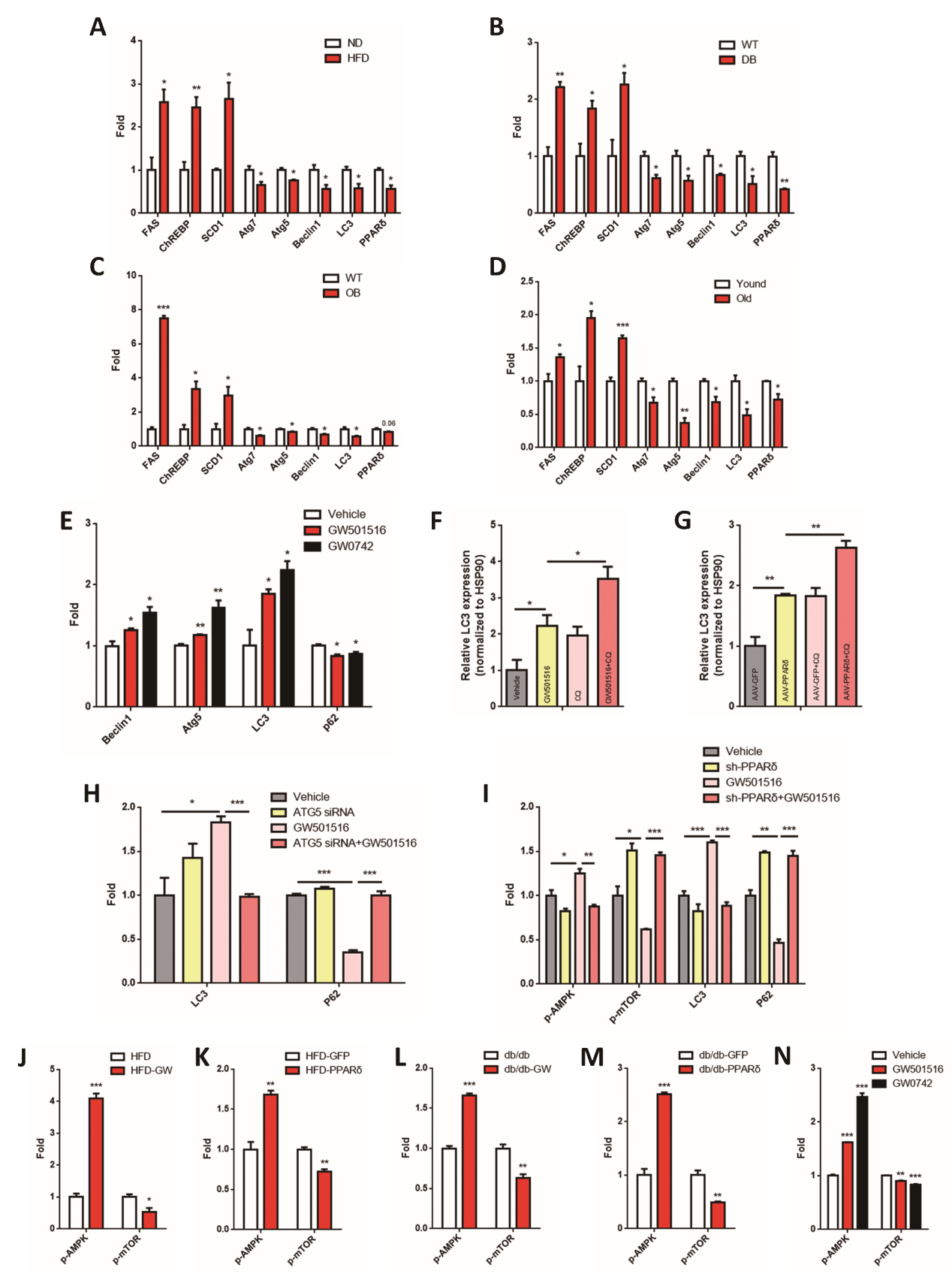


**Supplementary Figure 6** **Results of Western blots were quantified by AlphaView Software.** (A-D) quantified result of Fig 1. (E) quantified result of Fig 2. (F) quantified result of Fig 3. (G) quantified result of Fig 4. (H) quantified result of Fig 6. (I) quantified result of Fig 7. (J-N) quantified result of Fig 8. Data are expressed as the mean ± SEM of 3 independent experiments. *P < 0.05, **P < 0.01, ***P < 0.001.


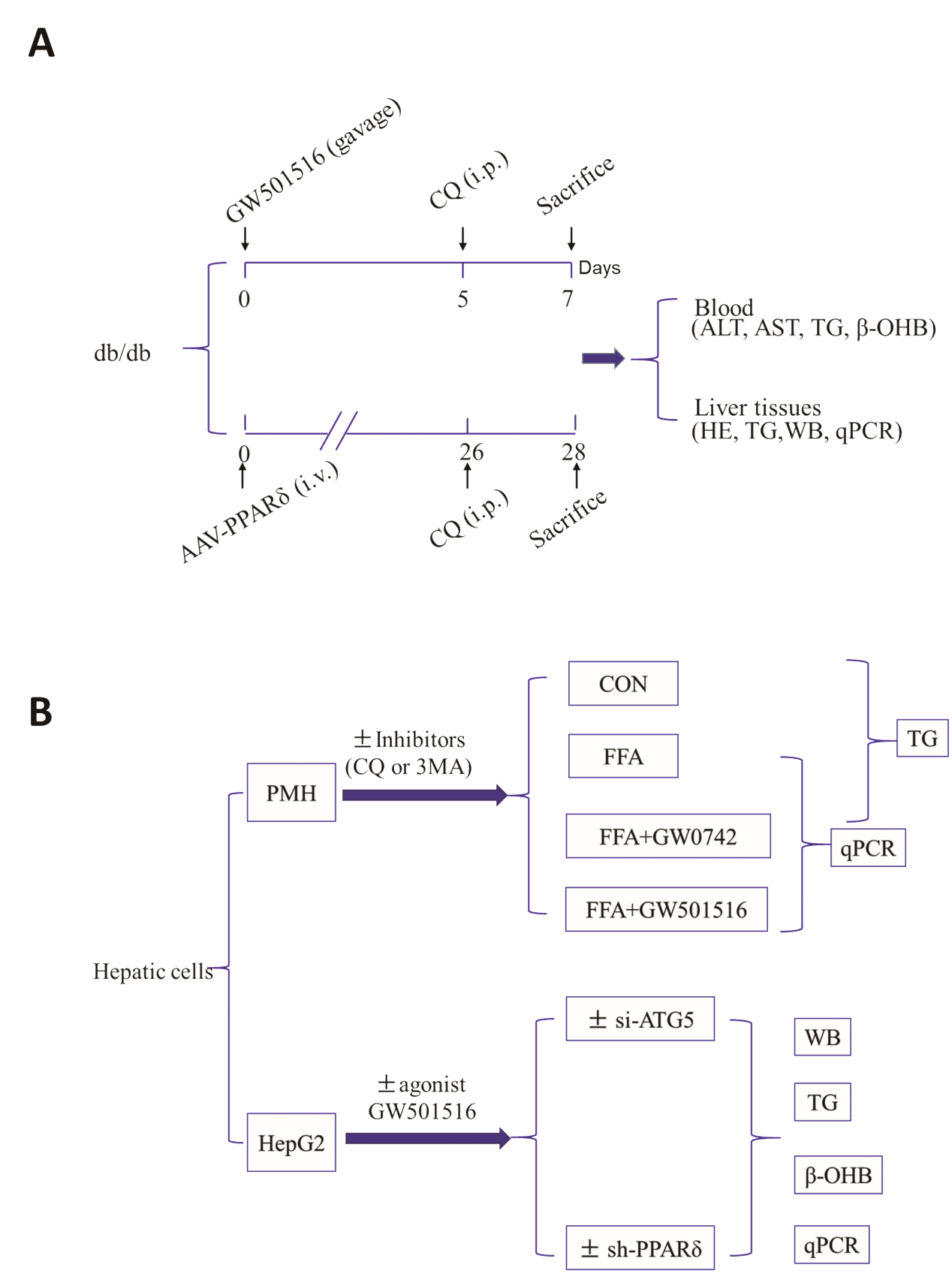


**Supplementary Figure 7** **The schematic representation.** (A) Animals treatment. (B) Cells treatment.
